# Low Light Availability Reduces the Subsurface Sediment Carbon Content in *Halophila beccarii* From the South China Sea

**DOI:** 10.3389/fpls.2021.664060

**Published:** 2021-06-07

**Authors:** Chanaka Premarathne, Zhijian Jiang, Jialu He, Yang Fang, Qiming Chen, Lijun Cui, Yunchao Wu, Songlin Liu, Zhao Chunyu, Prabath Vijerathna, Xiaoping Huang

**Affiliations:** ^1^Key Laboratory of Tropical Marine Bio-Resources and Ecology, South China Sea Institute of Oceanology, Chinese Academy of Sciences, Guangzhou, China; ^2^University of Chinese Academy of Sciences, Beijing, China; ^3^Southern Marine Science and Engineering Guangdong Laboratory, Guangzhou, China; ^4^Innovation Academy of South China Sea Ecology and Environmental Engineering, Chinese Academy of Sciences, Guangzhou, China; ^5^College of Resources, Environment and Planning, Dezhou University, Dezhou, China; ^6^Faculty of Science, University of Colombo, Colombo, Sri Lanka

**Keywords:** seagrass, light availability, vegetative carbon, sediment carbon, *Halophila beccarii*

## Abstract

Eutrophication, dredging, agricultural and urban runoffs, and epiphyte overgrowth could reduce light availability for seagrass. This may affect “blue carbon” stocks in seagrass beds. However, little research is available on the effect of light intensities on carbon sequestration capacity in seagrass beds, especially small-bodied seagrasses. The dominant seagrass *Halophila beccarii*, a vulnerable species on the IUCN Red List, was cultured in different light intensities to examine the response of vegetation and sediment carbon in seagrass beds. The results showed that low light significantly reduced leaf length and above-ground biomass, while carbon content in both above-ground and below-ground tissues were not affected. Low light reduced both the above-ground biomass carbon and the total biomass carbon. Interestingly, while under saturating light conditions, the subsurface and surface carbon content was similar, under low light conditions, subsurface sediment carbon was significantly lower than the surface content. The reduction of subsurface sediment carbon might be caused by less release flux of dissolved organic carbon from roots in low light. Taken together, these results indicate that reduced light intensities, to which these meadows are exposed to, will reduce carbon sequestration capacity in seagrass beds. Measures should be taken to eliminate the input of nutrients on seagrass meadows and dredging activities to maintain the “blue carbon” storage service by enhancing light penetration into seagrass.

## Introduction

Seagrasses are marine flowering plants, found on all continents except Antarctica (Hemminga and Duarte, 2000). Seagrass beds play a vital role in the ecosystem acting as one of the major primary producers with high productivity (Larkum et al., 2006; Valdez et al., 2020). Seagrasses provide multiple ecosystem services, including coastal protection, improved water quality through the uptake of nutrients, provision of nursery habitat, and carbon sequestration (Hemminga and Duarte, 2000; Fourqurean et al., 2012). Seagrass beds occupy only a very small fraction of the coastal vegetation but contribute to almost 25% of the annual carbon sequestration of the coastal zone, acting as a sink of CO_2_ (Duarte et al., 2013; Saderne et al., 2019). Organic carbon is not only stored in plant above- and below-ground compartments (i.e., shoots and roots), but is also stored in the sediment beneath seagrasses to a larger degree (Kennedy et al., 2010; Fourqurean et al., 2012; Jiang et al., 2017).

Underwater light intensity is one of the major factors that influence seagrass ecosystems, and seagrasses require nearly 11%–37% of the surface irradiance (Cussioli et al., 2020). Nevertheless, human activities in the catchment area and the coastal area can affect light availability for seagrass in the bottom habitats of the sea (Gattuso et al., 2006; Ralph et al., 2007). For example, eutrophication, dredging, agricultural and urban runoffs, and epiphyte overgrowth could reduce light availability for seagrasses (Ralph et al., 2007; York et al., 2015; Strydom et al., 2017; Yan et al., 2020). This undoubtedly inhibits seagrass photosynthesis, growth rate, and health status (Ralph et al., 2007). While most previous research has focused on the effect of light limitation on seagrass molecular and physiology (Dattolo et al., 2014; Schliep et al., 2015; Kumar et al., 2017; Davey et al., 2018; Griffiths et al., 2020), relatively little is known about the responses of carbon sequestration in seagrass beds (Serrano et al., 2014; Dahl et al., 2016).

The seagrass *Halophila beccarii* Asch is one of two species in the oldest lineage of seagrass distributed in the intertidal areas of the tropical Indo-Pacific region (Short et al., 2010; Aye et al., 2014; Jiang et al., 2020; Mishra and Apte, 2021). *H. beccarii* seems to be well adapted to the high light intensities when it gets exposed during low tides. *H. beccarii* often grows in river estuaries with large nutrient inputs. This results in higher epiphyte biomass attached to the leaves of *H. beccarii*, decreasing irradiance availability even further. Thus, *H. beccarii* has been declining at accelerating rates and is currently listed as a vulnerable species in the IUCN Red List of threatened species (Short et al., 2011; Jiang et al., 2020). Light limitation decreases seagrass carbon fixation and shoot density (Ralph et al., 2007; Ferguson et al., 2016), which might also reduce the amount of carbon available for root growth and root exudate formation (Jiang et al., 2018; Martin et al., 2018b). This may reduce the carbon stocks beneath seagrass meadows. While this has been confirmed by studying large-bodied species including *Posidonia* (Serrano et al., 2014) and *Thalassia* (Dahl et al., 2016), these effects have not been studied in small-bodied seagrass species, such as those in the *Halophila* genus.

Aiming to close some of these knowledge gaps, we conducted an indoor experiment to culture the seagrass *H. beccarii*, the dominant species in South China Sea, under different light intensities to examine the response of carbon storage in plant above- and below-ground compartments and associated sediments in seagrass beds. Two hypotheses were proposed. The first hypothesis was that low light reduces vegetative carbon stock due to decreased seagrass above-ground biomass. The second hypothesis was that carbon in the subsurface sediment (not including detritus) in seagrass beds was decreased by low light due to inhibited root growth and carbon allocation. Furthermore, we also estimated the change trend of vegetative carbon sequestration in *H. beccarii* beds in the South China Sea and globally caused by light limitation. The results obtained in the present study will undoubtedly enhance our understanding of the mechanisms controlling carbon storage in response to light. These will improve the management and conservation of these ecologically and economically important ecosystem engineers.

## Materials and Methods

### Plant Materials and Experimental Design

**FIGURE 1:**
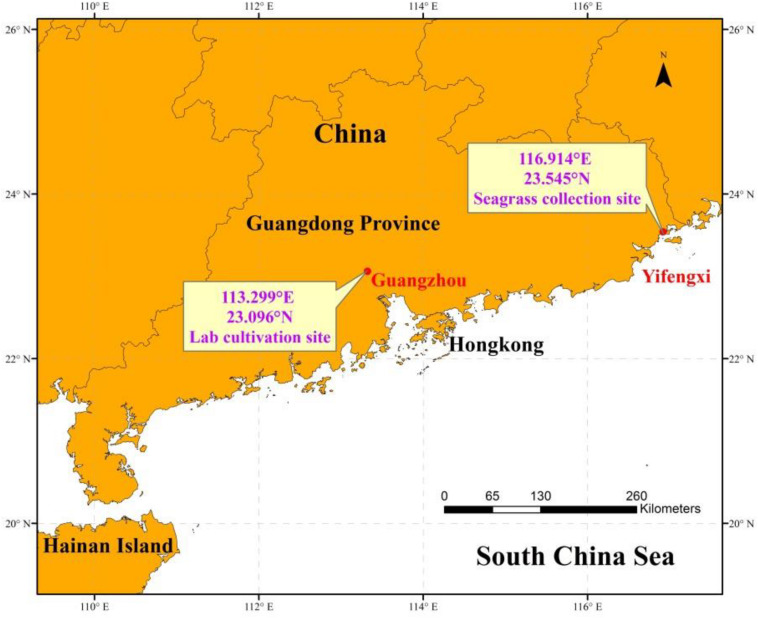
Location of seagrass collection and laboratory cultivation.

**FIGURE 2:**
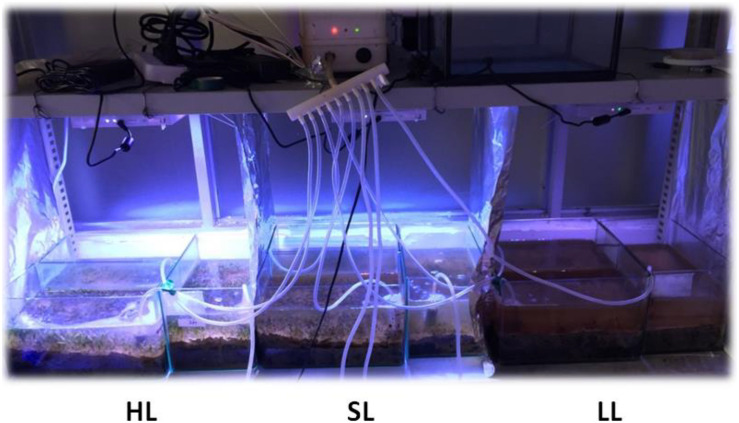
Experimental set-up of the laboratory treatments. SL represents the control saturating light (200 μmol photons/m^2^/s); LL represents low light (20 μmol photons/m^2^/s); and HL represents high light (600 μmol photons/m^2^/s). SL and HL were in the optimal light range between the minimum saturating light and the minimum inhibiting light.

*H. beccarii* plants were collected by hand in February 2019 with its natural sediment (6 cm sediment layer) using a smooth board (150 × 200 mm), at the intertidal zone of the monospecific seagrass bed in Yifengxi (116.903°E, 23.544°N; Figure 1), along the South China coast (Jiang et al., 2020). The shoot density of *H. beccarii* was about 7892 ± 744 shoots/m^2^ in the collection site. The leaf length, leaf width, and root length were 1.20 ± 0.05 cm, 0.26 ± 0.01 cm, and 1.97 ± 0.28 cm, respectively. Water turbidity at the collection site is relatively high, due to local eutrophication, agricultural, and urban runoffs. Following collection, plants were taken to the laboratory and placed within nine glass tanks (150 × 170 × 200 mm) with seawater. Based on preliminary relative electron transport rates (ETR) performed using rapid light curves (using the MINI PAM), its minimum saturating light was 177.3 ± 15.5 μmol photons/m^2^/s, approaching 200 μmol photons/m^2^/s. In the laboratory (113.299°E, 23.096°N, Guangzhou; Figure 1), seagrasses were cultured with natural seawater from the collection site at 200 μmol photons/m^2^/s for 1 week. This was for laboratory acclimation to minimize experimental error. After initial laboratory acclimation, three aquaria replicates were used for each of the three light treatments: the control saturating light (SL; 200 μmol photons/m^2^/s), high light (HL; 600 μmol photons/m^2^/s), and limited light (LL; 20 μmol photons/m^2^/s) irradiance. SL and HL were in the optimal light range between the minimum saturating light and the minimum inhibiting light. The average air temperature and humidity in the room were 25°C and 60%, respectively. The seawater temperature, salinity, and pH were 20°C, 3, and 8.00, respectively. Pump velocity and air-stone flow rate were kept the same across all aquaria to ensure effective stirring of the water body and gaseous diffusion (Figure 2). After 1 month of treatment (Supplementary Figure 1), seagrass and sediment were collected for measuring morphology (leaf length, leaf width, and root length), biomass of seagrass living tissues and detritus above the surface sediment, and nutrients and stable carbon isotope values of seagrass and sediment. Seagrass plants from five different places in each tank were collected for measuring leaf length, leaf width, and root length. Seagrass mature leaves were selected for determination. Sediment (not including detritus) of 6 cm was sampled with a modified syringe (the diameter was 29.5 mm, Supplementary Figure 2) and cut into two layers denoting the surface layer and subsurface layer.

The seagrass leaf length, leaf width, and root length were measured using a Vernier caliper. Seagrasses were carefully retrieved, separated into above-ground and below-ground tissue compartments, and subsequently dried at 60°C for 72 h until a constant weight was achieved. Seagrasses were then homogeneously powdered. The total carbon and nitrogen levels of seagrasses were analyzed using an Elementary Analyzer (Flash EA 3000 Thermo Fisher Scientific, Milan, Italy).

The sediment samples were freeze-dried, and sieved through a 500 μm screen to remove coarse materials, which were weighed so their mass could be accounted for in later calculations. Samples were ground and homogenized with a mortar and pestle. All samples were stored in a desiccator prior to analysis. The concentrations of sediment carbon were determined with a CHN analyzer (Elementar, Vario EL-III, Germany). We did not acidify sediment to remove inorganic carbon, since the sediment is mainly composed of organic carbon. δ^13^C isotopes in seagrass and sediments were analyzed using a continuous-flow isotope-ratio mass spectrometer (Delta V Advantage, Thermo Fisher Scientific, Waltham, MA, United States). δ^13^C = (R_*sample*_/R_*standard*_-1) × 1000, where R is the ratio of ^13^C/^12^C. The reference standard for carbon was Vienna PeeDee Belemnite.

We estimated the total vegetative carbon stock of seagrass using the following equations (Howard et al., 2014; Lian et al., 2018):

Vegetative component carbon pool (Mg C/ha) = Carbon content (kg C/m^2^) × (Mg/1,000 kg) × (10,000 m^2^/ha).

Likewise, the total vegetative nitrogen stock of seagrass was also calculated.

### Statistical Analysis

Statistical analysis was conducted using Minitab 17.0 Statistical software. The means and standard errors of all variables were calculated, and all the data were first tested to determine whether the assumptions of homogeneity and normality were met. Where these assumptions were not met, the raw data were transformed and a further statistical analysis was conducted using the dataset that fulfilled the assumptions. One-way ANOVA followed by Tukey’s multiple comparisons tests were performed to determine whether the parameters of seagrass were significantly different among light treatments. Differences between mean values were considered to be significant at a probability of 5% (*p* < 0.05). Otherwise, Welch’s t test was performed followed by Dunnett’s T3’s multiple comparisons tests for determining the significance (*p* < 0.05) (Giannios and Casanova, 2021) of seagrass parameters among light treatments. Two-way ANOVA was performed to investigate the significant difference of C and ^13^C/^12^C in sediments with respect to light stress and sediment layer.

## Results

### Seagrass Morphology and Biomass

**FIGURE 3:**
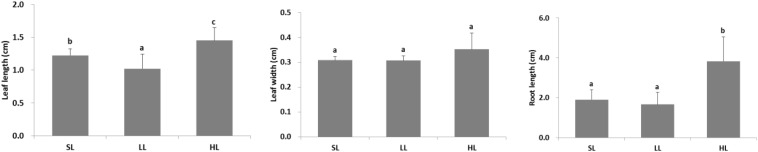
Response of seagrass morphology to light treatments. Saturating light: SL, 200 μmol photons/m^2^/s; low light: LL, 20 μmol photons/m^2^/s; high light: HL, 600 μmol photons/m^2^/s. SL and HL were in the optimal light range between the minimum saturating light and the minimum inhibiting light. Different letters on the bars indicate mean values for a particular light condition that significantly differed at (*p* < 0.05) (Mean ± SD, *n* = 15) analyzed by one-way ANOVA.

**FIGURE 4:**
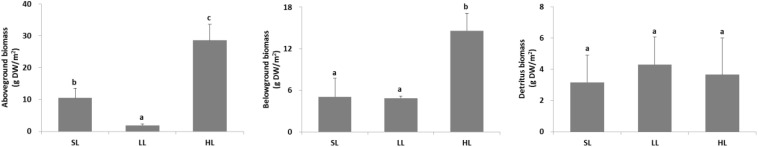
Response of seagrass biomass to light treatments. Saturating light: SL, 200 μmol photons/m^2^/s; low light: LL, 20 μmol photons/m^2^/s; high light: HL, 600 μmol photons/m^2^/s. SL and HL were in the optimal light range between the minimum saturating light and the minimum inhibiting light. Different letters on the bars indicate mean values for a particular light condition that significantly differed at (*p* < 0.05) (Mean ± SD, *n* = 3) analyzed by one-way ANOVA.

The seagrass morphology is depicted in Figure 3. A significant difference was found for leaf length and root length (Table 1). A declined trend was observed for leaf length and root length along the decreased light irradiance. Simultaneously, there were differences for biomass of above-ground and below-ground tissues among light treatments (Table 2). Biomass of above-ground and below-ground tissues both decreased along with decreased light irradiance (Figure 4 and Supplementary Figure 1). Especially, above-ground biomass of SL and HL were about 5 times and 15 times of that of LL, respectively. Interestingly, the detritus biomass was higher in LL than in SL and HL, although there was no considerable difference.

**TABLE 1:** Statistical analysis of effects of different light intensities on seagrass parameters.

**Variable**	**Statistic (asymptotically F distributed)**	**df1**	**df2**	***P***
Leave length	15.71	2	24.58	<0.05
Leave width	3.29	2	24.83	0.054
Root length	19.22	2	25.97	<0.05
Above-ground carbon	2.90	2	3.46	0.182
Below-ground carbon	3.14	2	3.04	0.182
Detritus carbon	0.87	2	2.77	0.509

*Welch test, *P* < 0.05 at significant level.*

**TABLE 2:** Statistical analysis of effects of different light intensities on seagrass parameters.

**Variable**	**df**	***F***	***P***
Above-ground biomass	2	47.60	<0.01
Below-ground biomass	2	20.27	<0.01
Detritus biomass	2	0.25	0.787
Above-ground nitrogen	2	13.74	<0.01
Below-ground nitrogen	2	23.30	<0.01
Detritus nitrogen	2	1.94	0.223
Above-ground biomass carbon	2	78.07	<0.01
Below-ground biomass carbon	2	4.18	0.073
Detritus biomass carbon	2	0.43	0.668
Total biomass carbon	2	29.44	<0.01
Above-ground biomass nitrogen	2	70.41	<0.01
Below-ground biomass nitrogen	2	0.72	0.526
Detritus biomass nitrogen	2	0.53	0.611
Total biomass nitrogen	2	31.71	<0.01
Above-ground δ^13^C	2	477.02	<0.01

*P < 0.05 (significant); *P* < 0.01 (highly significant).*

### Seagrass Carbon and Nitrogen

**FIGURE 5:**
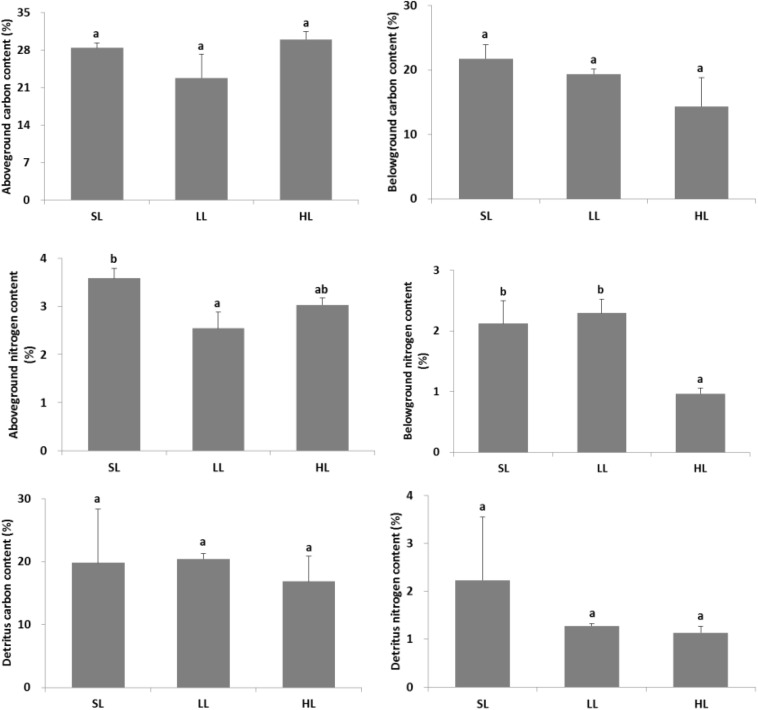
Response of seagrass nutrients to light treatments. Saturating light: SL, 200 μmol photons/m^2^/s; low light: LL, 20 μmol photons/m^2^/s; high light: HL, 600 μmol photons/m^2^/s. SL and HL were in the optimal light range between the minimum saturating light and the minimum inhibiting light. Different letters on the bars indicate mean values for a particular light condition that significantly differed at (*p* < 0.05) (Mean ± SD, *n* = 3) analyzed by one-way ANOVA.

**FIGURE 6:**
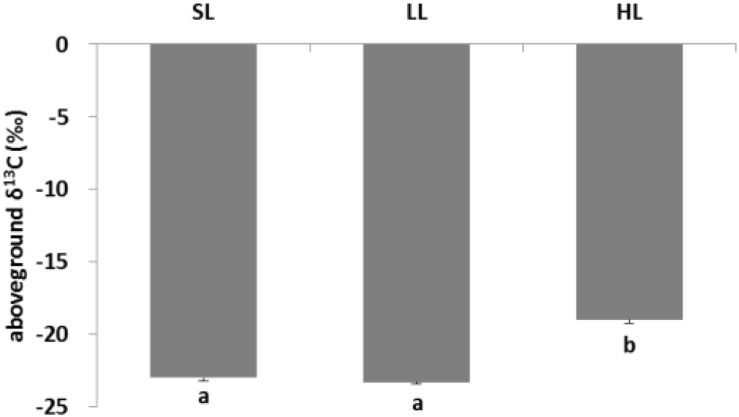
Response of seagrass stable isotope carbon to light treatments. Saturating light: SL, 200 μmol photons/m^2^/s; low light: LL, 20 μmol photons/m^2^/s; high light: HL, 600 μmol photons/m^2^/s. SL and HL were in the optimal light range between the minimum saturating light and the minimum inhibiting light. Different letters on the bars indicate mean values for a particular light condition that significantly differed at (*p* < 0.05) (Mean ± SD, *n* = 3) analyzed by one-way ANOVA.

The response of seagrass nutrients to light treatments is depicted in Figure 5. There was no significant difference for seagrass carbon, while there was a marked difference for nitrogen in both above-ground and below-ground tissues (Tables 1, 2). Carbon and nitrogen were the lowest in the above-ground tissue in the LL treatment. Interestingly, carbon content under HL was the highest in the above-ground tissue, while carbon content exhibited the lowest levels in the below-ground tissue. Simultaneously, detritus carbon and nitrogen, as well as nitrogen content in the below-ground tissues were the lowest under HL treatment (Figure 5). Furthermore, the difference of δ^13^C content in the above-ground tissues was significant among light treatments (Table 2), with a higher value in the HL treatment (Figure 6).

**FIGURE 7:**
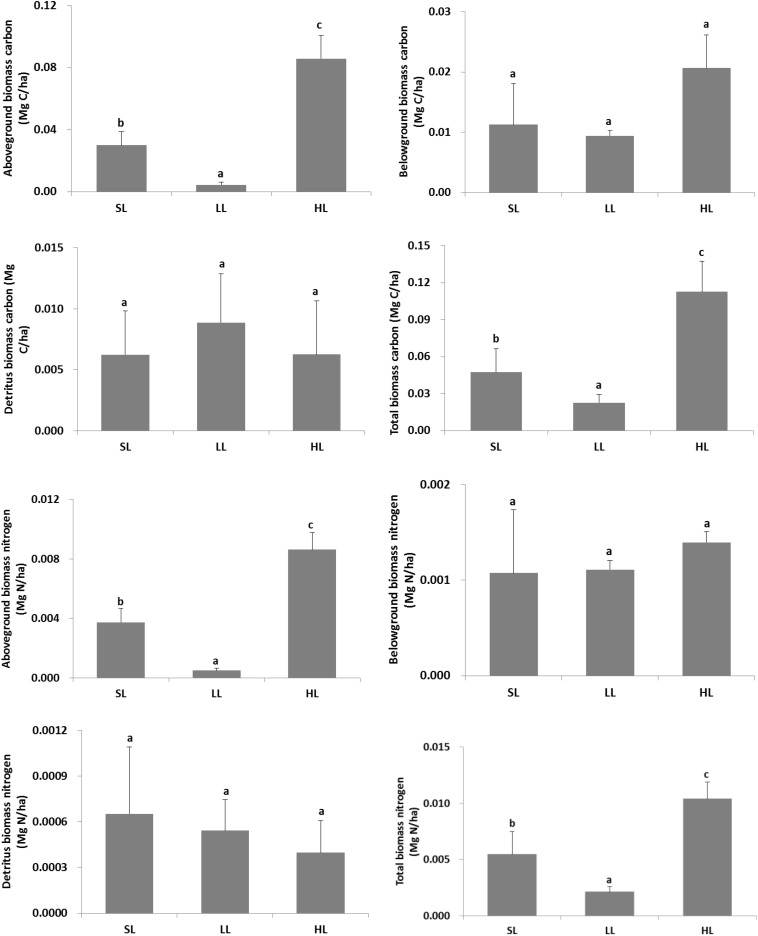
Response of vegetative carbon and nitrogen stock to light treatments. Total biomass included living above-ground and below-ground tissues, and detritus above the surface sediment. Saturating light: SL, 200 μmol photons/m^2^/s; low light: LL, 20 μmol photons/m^2^/s; high light: HL, 600 μmol photons/m^2^/s. SL and HL were in the optimal light range between the minimum saturating light and the minimum inhibiting light. Different letters on the bars indicate mean values for a particular light condition that significantly differed at (*p* < 0.05) (Mean ± SD, *n* = 3) analyzed by one-way ANOVA.

The changes of plant carbon and nitrogen stock in response to light treatments are displayed in Figure 7. A significant difference was found for the living above-ground and total plant biomass carbon and nitrogen stock (Table 2). Biomass carbon in the above-ground, below-ground tissues, and combined plant compartments (i.e., the entire plant biomass) all exhibited a decreased trend along with decreased light intensities. A similar trend was also found for the living above-ground and total biomass nitrogen. Furthermore, the detritus biomass carbon was also higher in LL than in the other two light intensities.

### Sediment Carbon

**FIGURE 8:**
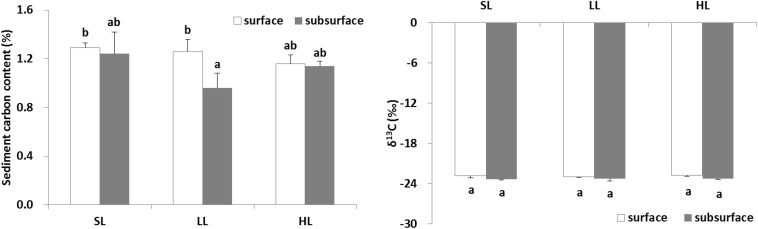
Response of sediment carbon to light treatments. Saturating light: SL, 200 μmol photons/m^2^/s; low light: LL, 20 μmol photons/m^2^/s; high light: HL, 600 μmol photons/m^2^/s. SL and HL were in the optimal light range between the minimum saturating light and the minimum inhibiting light. Different letters on the bars indicate mean values for a particular light condition that significantly differed at (*p* < 0.05) (Mean ± SD, *n* = 3) analyzed by two-way ANOVA.

The effects of light intensities on sediment carbon are shown in Figure 8. The carbon content in the surface and subsurface of the sediments was found to be similar in the SL and HL treatment, while the carbon concentration in the subsurface sediment was significantly lower than in the surface sediment in the LL treatment (Table 3). It was similar for the sediment δ^13^C among the light treatments and between layers.

**TABLE 3:** Statistical analysis of effects of different light intensities on sediment parameters.

**Parameters**	**C%**						**^13^C/^12^C**				
**Source**	**DF**	**SS**	**MS**	***F***	***P***		**DF**	**SS**	**MS**	***F***	***P***
Light	2	0.075	0.038	3.54	0.062		2	0.031	0.016	0.29	0.75
Sediment layer	1	0.067	0.067	6.34	0.027		1	0.768	0.768	14.44	0.003
Light*sediment layer	2	0.074	0.037	3.49	0.064		2	0.036	0.018	0.33	0.723
Error	12	0.127	0.011				12	0.638	0.053		
Total	17	0.344					17	1.473			
R-Sq	R-Sq = 62.96%					‘	R-Sq = 56.67%				

## Discussion

The decrease in light availability is considered as the main anthropogenic disturbance to seagrass beds, causing lower carbon burial capacity (Schmidt et al., 2012; Dahl et al., 2016). The present study provided an opportunity to examine variation in plant and sediment carbon sequestration in seagrass beds across a wide range of light. The findings demonstrated that exposure to low light reduced vegetative carbon stock and subsurface sediment carbon in seagrass beds.

### Low Light Decreased Vegetative Carbon Stock in Seagrass Beds

Morphological plasticity allow seagrasses to withstand changes in light availability (Ralph et al., 2007; Ferguson et al., 2016). The present study demonstrated that low light significantly reduced leaf length, above-ground biomass, and leaf densities (Supplementary Figure 3). Seagrasses are more sensitive to light reduction since high light is required to maintain a large quantity of non-photosynthetic tissue (Erftemeijer and Lewis Iii, 2006). Meanwhile, leaf density of *H. beccarii* (Ismail, 2014) and shoot density of *Zostera muelleri* (Ferguson et al., 2016) were also reduced by light limitation. Furthermore, light reduction even resulted in complete mortality for *Halophila ovalis* in a turbid environment (Yaakub et al., 2014). In contrast, increasing leaf length or area could allow seagrasses to acclimate to low light climates (Longstaff and Dennison, 1999; Collier et al., 2009; Yaakub et al., 2014; Azcárate-García et al., 2020; Winters et al., 2020). The difference might be caused by the fact that the low light condition in the present study was too limited to maintain a positive carbon balance for *H. beccarii*. Furthermore, low light decreased the energy for generation of ATP for both carbon fixation and HCO_3_^–^ uptake (Ow et al., 2016). Low light induced less nitrogen content in the above-ground tissue, which might result in lower chlorophyll synthesis (Wen et al., 2019). This undoubtedly reduced seagrass carbon fixation. Similarly, low light also reduced the leaf starch of *H. ovalis* (Strydom et al., 2017). Thus, it would decrease the transport of photosynthetically derived non-structural carbohydrates to the root-rhizome system, leading to lower production of below-ground tissues (Duarte and Chiscano, 1999). Meanwhile, a reduction in non-structural carbohydrates also depleted carbon storage reserves that could be used when exposed to further stressors and might therefore reduce seagrass meadow resilience in the future (Alcoverro et al., 2001; Krause-Jensen et al., 2021). The decrease of biomass of above-ground and below-ground tissues also allowed light-limited plants to reduce carbon demands for respiration and maintain overall carbon balance (Lee et al., 2007). High shading also resulted in 45% lower carbon content in the below-ground tissue compared to control treatment (Dahl et al., 2016). Furthermore, detritus biomass was higher in low light, indicating that low light not only inhibited seagrass growth, but also induced leaf senescence to produce higher leaf detritus (York et al., 2013).

**FIGURE 9:**
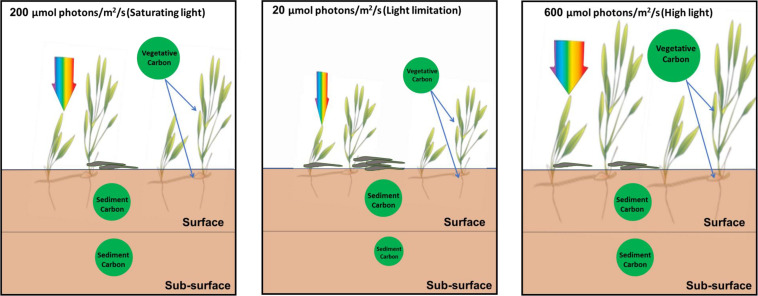
Schematic pictures of the effects of light reduction on the carbon sequestration in seagrass beds.

The biomass carbon and nitrogen stocks of living above-ground tissue were significantly reduced under lower light conditions compared to saturating light. Especially, the total biomass carbon stock of seagrass plants under low light was about half of that under saturating light, indicating plant carbon stock decreased to a great extent under limiting light (Figure 9).

Based on the area (about 1158.74 ha) of *H. beccarii* in the South China Sea (Jiang et al., 2017, 2020; Huang et al., 2019), if the light availability for all *H. beccarii* beds was reduced to 20 μmol photons/m^2^/s from 200 μmol photons/m^2^/s by anthropogenic activities, its vegetative carbon and nitrogen stock would decrease by 28.74 Mg C and 3.86 Mg N, respectively. The global vegetative carbon and nitrogen stock of *H. beccarii* (the global area was estimated to be less than 2000 km^2^ (Short et al., 2010), we calculated it using 2000 km^2^) would also decrease by 4958.69 Mg C and 665.50 Mg N, respectively. Therefore, light limitation caused by anthropogenic activities would not only reduce the carbon sequestration in biomass, but also damage the ecological service of filtering the nutrients and bacteria within the water column (Lamb et al., 2017), a service that is estimated at 10 million $/year (Campagne et al., 2015) for this vast area covered by meadows.

In addition, higher δ^13^C was observed in the above-ground tissue under HL, which might be induced by increased uptake of ^13^C from the external C source (Grice et al., 1996). Interestingly, high light intensity exhibited the lowest carbon and nitrogen in the below-ground tissue of *H. beccarii*, while the biomasses of above-ground and below-ground tissues were the highest. This indicated that below-ground growth was enhanced by high light to dilute the nutrient content (Peralta et al., 2000). Furthermore, seagrasses in high light treatments were also shown to respond to these low nutrient conditions by increasing their root biomass in order to try and get more nutrients from the sediment (Abal et al., 1994; Grice et al., 1996).

### Low Light Reduced Subsurface Sediment Carbon Contents Compared to Surface Sediment

The change of seagrass productivity and biomass caused by anthropogenic activities might result in the decreased flow of organic carbon sequestrated in the sediment (Dahl et al., 2016; Jiang et al., 2018). The release of root exudates might be of particular importance in subsurface sediment systems (Zhai et al., 2013). About 11% of total fixed carbon in *Halodule wrightii* was exuded into the sediment (Moriarty et al., 1986). Interestingly, the present study demonstrated that sediment carbon contents between surface and subsurface layers were similar in both saturating and high light, while subsurface sediment carbon was significantly lower (about 24%) than surface sediment carbon (Figure 8) under low light irradiance (Figure 9). This indicated that low light reduced subsurface sediment carbon contents compared to surface sediment. Meanwhile, depth also explained the carbon content in seagrass sediment, with lower carbon contents at deeper sites attributed to decreased light penetration (Serrano et al., 2014; Samper-Villarreal et al., 2016). Low light reduced root length and biomass (Martin et al., 2018b). The reduction of root biomass would decrease the flux of root exudation of dissolved organic carbon into sediment (Jiang et al., 2018). This undoubtedly lowered subsurface sediment carbon content. Similarly, the above-ground light reduction also invoked a cascade of changes from alterations in root exudation to a decrease in putative beneficial microorganisms (Martin et al., 2018a). However, no significant linear relationship between *Zostera marina*-dissolved organic carbon exudation and light treatment was observed (Kaldy, 2012). The difference might be caused by the fact that dissolved organic carbon exudation rates might be correlated to seagrass species-specific attributes. Nutrient enrichment also significantly reduced the sediment organic carbon content in a 6-21 cm layer around the seagrass root system of *Thalassia hemprichii* and *Enhalus acoroides* in Xincun Bay (Jiang et al., 2018). Therefore, eutrophication weakened subsurface sediment carbon sequestration by lowering light availability or enhancing toxic effect of nutrients on seagrasses.

### Ecological Significance and Conclusion

The present study found that the reduction of light availability for seagrass caused by eutrophication and agricultural and urban runoff decreased the vegetative carbon of *H. beccarii* and subsurface sediment carbon content in seagrass beds (Figure 9). Meanwhile, low light availability also decreased the canopy complexity of *H. beccarii*. This would most likely trap less allochthonous organic matter in the seagrass canopy and be less efficient in the deposition of fine-grained particles, and thus might also have negative effects on the carbon sequestration capacity of *H. beccarii* (Agawin and Duarte, 2002; Hendriks et al., 2008; Samper-Villarreal et al., 2016; Gullström et al., 2018). The carbon stored in the sediment in seagrass beds is vulnerable to export and remineralization if shoot densities are reduced or seagrass cover is lost due to reduced irradiance (Pendleton et al., 2012; Dahl et al., 2016). *H. beccarii* is primarily distributed in river mudflats with large nutrient inputs in the South China Sea (Jiang et al., 2017, 2020). To ensure continued productivity and maintain the carbon sequestration capacity in *H. beccarii* beds in the future, the nutrient inputs and dredging activities should be reduced to improve water quality to enhance light penetration. In addition, removal of the epiphytes on seagrass leaves by using the combination of an acid treatment with moderate scraping without seriously damaging leaf substratum (Dauby and Poulicek, 1995) would also be a feasible measure to enhance carbon sequestration in seagrass beds.

The present study showed that light availability influenced the primary production as shown in the decreased above-ground biomass in the low light treatments. If above-ground biomass is reduced, photosynthesis will be impacted and seagrasses might as a response exude less photosynthates from their roots into the sediment which will in turn impact microbial communities (Ding et al., 2015; Dahl et al., 2016; Jiang et al., 2018; Martin et al., 2018a). Those microbial communities are essential in creating the precursors of stable organic matter which they do by using their host’s root exudates (Cotrufo et al., 2013; Kallenbach et al., 2016). So if the seagrass host cannot supply their microbial communities with sufficient root exudates the carbon sequestration will be negatively affected. Therefore, future research should focus on examining the effect of low light on seagrass root exudates composition and rhizosphere bacterial communities, as well as their influence on sediment carbon transformation processes.

## Data Availability Statement

The raw data supporting the conclusions of this article will be made available by the authors, without undue reservation.

## Author Contributions

ZJ and XH designed the study. CP, ZJ, JH, YF, QC, LC, YW, SL, ZC, and PV performed the experiments or analyzed the data. CP, ZJ, and XH wrote the manuscript. All authors contributed to the article and approved the submitted version.

## Conflict of Interest

The authors declare that the research was conducted in the absence of any commercial or financial relationships that could be construed as a potential conflict of interest.
