# Supplementary material for: Low Light Availability Reduces the Subsurface Sediment Carbon Content in Halophila beccarii From the South China Sea
Source: Front Plant Sci. 2021 Jun 7;12:664060. doi: 10.3389/fpls.2021.664060 (PMC8215720; doi:10.3389/fpls.2021.664060)

Fig S1 Picture of light treatment experiment in the end. Saturating light: SL, 200 μmol photons/m^2/s; Low light: LL, 20 μmol photons/m^2/s; High light: HL, 600 μmol photons/m^2/s. SL and HL was in the optimal light range between the minimum saturating light and the minimum inhibiting light.

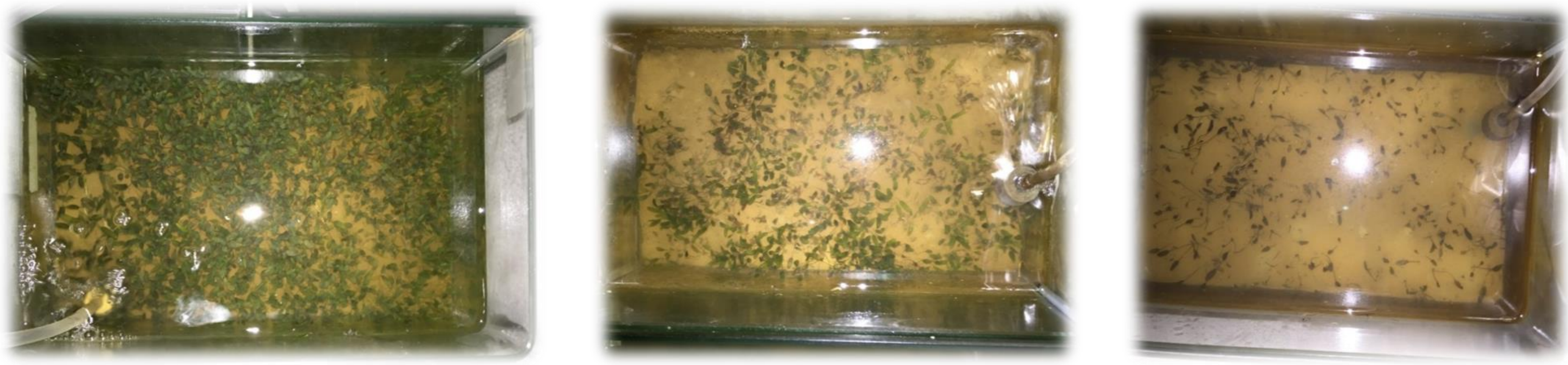

HL **SL** **LL**

Fig S2 Schematic picture of modified syringe used for collecting sediment samples

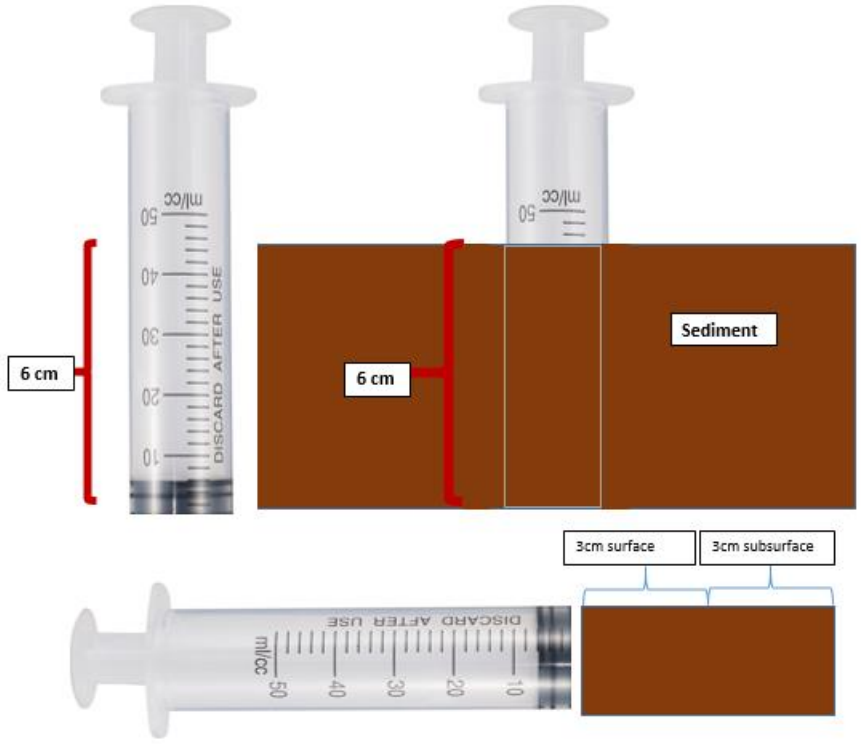

Fig S3 Response of seagrass leaf density to light treatments. Saturating light: SL, 200 μmol photons/m^2/s; Low light: LL, 20 μmol photons/m^2/s; High light: HL, 600 μmol photons/m^2/s. SL and HL was in the optimal light range between the minimum saturating light and the minimum inhibiting light.

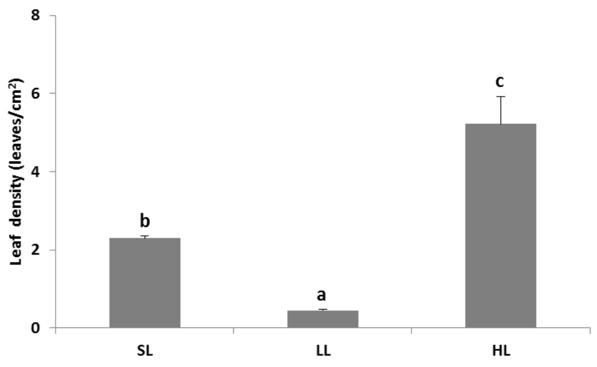

Supplement: Supplementary file 1 [file Data_Sheet_1.pdf]
